# First Activation of the WHO Emergency Medical Team Minimum Data Set in the 2019 Response to Tropical Cyclone Idai in Mozambique

**DOI:** 10.1017/S1049023X22001406

**Published:** 2022-12

**Authors:** Tatsuhiko Kubo, Odgerel Chimed-Ochir, Matchecane Cossa, Isse Ussene, Yoshiki Toyokuni, Yui Yumiya, Ryoma Kayano, Flavio Salio

**Affiliations:** 1. Japan Disaster Relief Expert Team for Mozambique Cyclone Idai Response 2019; 2. Department of Public Health and Health Policy, Graduate School of Biomedical and Health Sciences, Hiroshima University, Hiroshima, Japan; 3. National Program of Surgery, Ministry of Health of Mozambique, Maputo, Mozambique; Avenida Eduardo Mondlane, Maputo, Mozambique; 4. National Hospital Organization Headquarters DMAT Secretariat MHLW Japan, Tokyo, Japan; 5. World Health Organization Centre for Health Development (WHO Kobe Centre), Kobe, Japan; 6. Emergency Medical Teams, World Health Organization, Geneva, Switzerland

**Keywords:** emergency medical team, emergency medical team minimum data set, Idai cyclone, Mozambique

## Abstract

**Introduction:**

During a disaster, comprehensive, accurate, timely, and standardized health data collection is needed to improve patient care and support effective responses. In 2017, the World Health Organization (WHO) developed the Emergency Medical Team (EMT) Minimum Data Set (MDS) as an international standard for data collection in the context of disasters and public health emergencies. The EMT MDS was formally activated for the first time in 2019 during the response to Cyclone Idai in Mozambique.

**Study Objective:**

The aim of this study was to analyze data collected through the EMT MDS during Cyclone Idai of 2019 and to identify the benefits of and opportunities for its future use.

**Methods:**

The EMT MDS was used for data collection. All 13 international EMTs deployed from March 27 through July 12 reported data in accordance with the EMT MDS form. The collected data were analyzed descriptively.

**Results:**

A total of 18,468 consultations, including delivery of 94 live births, were recorded. For children under-five and those five-years and older, the top five reasons for consultation were minor injuries (4.5% and 10.8%, respectively), acute respiratory infections ([ARI] 12.6% and 4.8%, respectively), acute watery diarrhea (18.7% and 7.7%, respectively), malaria (9.2% and 6.1%, respectively), and skin diseases (5.1% and 3.1%, respectively). Non-disaster-related health events accounted for 84.7% of the total health problems recorded. Obstetric care was among the core services provided by EMTs during the response.

**Conclusion:**

Despite of challenges, the EMT MDS reporting system was found to support the responses and coordination of EMTs. The role of the Mozambican Ministry of Health (MOH), its cooperation with EMTs, and the dedicated technical support of international organizations enabled its successful implementation.

## Introduction

The world has witnessed a ten-fold increase in the number of natural disasters since 1960.^1^ A disaster is defined as a situation or event that overwhelms the local capacity, necessitating a request for national- or international-level assistance.^2^ During an emergency or disaster, affected patients are treated by Emergency Medical Teams (EMTs), which are groups of health professionals that include doctors, nurses, paramedics, support workers, and logisticians. Such teams can be governmental (both civilian and military) and non-governmental, and they can be further classified as national or international, depending on the area of response. Considering the significant surge of medical demand and the variety of services provided by EMTs, information from deployed EMTs must be gathered and analyzed to enable the coordination and decision making needed for a timely and effective response.^3^

The Emergency Medical Team Coordination Cell (EMTCC) provides a common technical platform for coordinating EMTs.^3^ The EMTCC is set and managed as an internal entity of the affected government and its Ministry of Health (MOH) with assistance from the World Health Organization (WHO; Geneva, Switzerland) and other partners, as required. The gathering of information from EMTs is critical for the ability of the EMTCC to fulfill its coordination role, but only a few countries have a reporting template to be used by EMTs and health facilities during an emergency response. Furthermore, EMTs mobilized by a variety of organizations arrive on site with their own medical forms and/or reporting systems, rendering it difficult to compile information and use it to allocate resources, deploy emergency teams, and predict health care needs during disasters.^4,5^ In 2017, to address this gap, a technical working group of the WHO defined a package of essential data items for EMT reporting: the Emergency Medical Team Minimum Data Set (EMT MDS) and its daily reporting form. The EMT MDS comprises four components and was developed to be simple and quick to complete while providing a picture of the emergency situation and helping guide operational resource management at the level of the EMTCC/MOH (hereinafter EMTCC).

In March 2019, Mozambique was hit by Cyclone Idai, which has been recognized as one of the deadliest cyclones on record for the Southern Hemisphere. The cyclone made landfall in the city of Beria in the province of Sofala and caused a total death toll of more than 750 people.^6^ Flooding caused by the cyclone displaced thousands of people and severely disrupted health services, including chronic disease treatment and prenatal health care.^7^ Upon a request for assistance from the Government of Mozambique, international EMTs were dispatched and the EMT MDS was activated for the first time in a large international-scale response. Here, the data collected through the EMT MDS during Cyclone Idai of 2019 were analyzed and the benefits of and opportunities were identified for its future use.

## Methods

### EMT MDS Daily Reporting Form

The EMT MDS daily reporting form comprises a total of 85 items divided into four main sections: Team Information (14 items, from A through N); Daily Summary (six items, from O through T); MDS Statistics (No. 1-50: demographic information; health events, including trauma, infectious disease, additional, emergency, and other key diseases; procedures and outcomes; and context in relation to the event, protection issues); and Needs and Risks (No. 51-65). To complete the form, EMT staff must directly type information into the “Team Information,” “Daily Summary,” and “Needs and Risks” sections. The MDS Statistics section is completed from pre-populated boxes based on the result of each consultation. All patient data were collected anonymously. The original EMT MDS daily reporting form and definitions of its items can be found in Supplementary Table 1 (available online only), and additional details on its use are available through its website.^8^

### Data Collection

Tropical Cyclone Idai made landfall near Beira City, Sofala Province, in central Mozambique on the night of March 14 to 15, 2019, and an international-scale humanitarian response, including EMTs, started soon after. Given its scale and the need to setup an ad-hoc surveillance system among the EMTs, the EMTCC of Mozambique officially announced the use of the MDS daily reporting form on March 31, 2019 and tasked all EMTs with daily reporting using this template. Considering the national context and burden of diseases, the EMTCC proposed the addition of a blank column at MDS No. 19 to capture information on malaria. The WHO and Japan Disaster Relief Expert Team provided technical assistance for the activation. All 13 international EMTs deployed to Mozambique from March 27 through July 12 reported data in accordance with the MDS form. All EMTs received the relevant forms and tools from the EMTCC.^8^

Each EMT aggregated their daily report by using a free Microsoft Excel (Microsoft Corp.; Redmond, Washington USA) tool called the MDS Maker, which was provided by the EMTCC. This generated comma-separated value (csv) files, which were emailed to the EMTCC. Some EMTs, such as Japan Disaster Relief and Team Rubicon, generated the csv file using a sophisticated IT system rather than the free Excel tool. The EMTs that did not have access to the internet physically delivered their daily reports to the EMTCC. Because some EMTs were unable to send csv files to the EMTCC, the MDS off site analysis support team from Japan assisted in converting all delivered reports into the standard csv format and remotely supported the data analysis. Because of the seven-hour time difference between Japan and Mozambique, the Japan team was able to work on data and send the converted csv files back to the EMTCC by the following morning in Mozambique. Daily summary statistics were generated using a tool called MDS Feedback Maker.

### Data Analysis

During the 110 days of the response, a total of 282 daily reports were sent by EMTs to the EMTCC. A descriptive analysis of the MDS statistics was conducted. According to the structure of the reporting form, the health events were analyzed based on two age groups: those under-five years and those five-years and over. Following a descriptive analysis of all health events, the five most frequent health events (minor injury, acute watery diarrhea, acute respiratory infections [ARI], malaria, and skin diseases) were selected and a detailed analysis was provided. The item “other diagnosis, not specified above” was removed from the analysis. In terms of response period, the total period was divided into 16 periods of one week each.

Pearson chi square test was used to examine the between-age-group differences of health consultations and specific health problems. Microsoft Excel and STATA v15.1 (StataCorp; College Station, Texas USA) were used for analysis.

### Ethical Review

Approval for ethical review was obtained from Hiroshima University (Hiroshima, Japan; approval number: E-2059).

## Results

A total of 282 daily reports generated using the EMT MDS form were collected from 13 EMTs during the period spanning March 27 through July 12, 2019.

**Figure 1.**
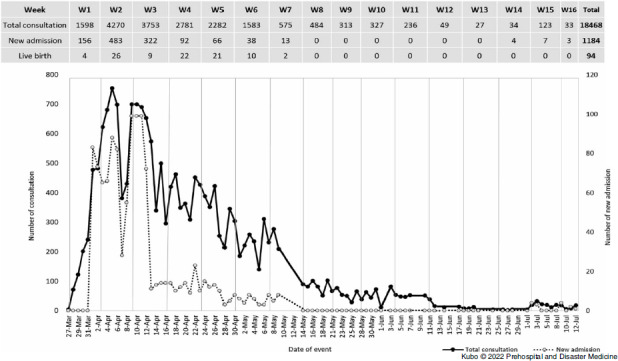
Total Number of Consultation and New Admission during Cyclone Idai.

From the “Daily Summary” section, the total number of consultations, new admissions, and live births were identified by period (Figure 1). During the reporting period, a total of 18,468 consultations, 1,184 new admissions, and 94 live births were reported. The highest numbers of consultations (4,270) and new admissions (483) occurred in Week 2 following EMT dispatch; the numbers of both gradually decreased thereafter. All live births recorded by EMTs happened within the first seven weeks of the reporting period.

Table 1 depicts an analysis of the MDS Statistics subsection entitled “Demographic.” In total, 17,098 consultations that included gender information were analyzed. Women had a higher percentage of consultations (55.7%) than men (44.3%). In terms of age distribution, patients aged 18 to 64 accounted for 65.9% of total consultations, while those over 65 accounted for only 6.1%.

**Table 1.** Demographic Characteristics of Health Consultation during Cyclone Idai

Age	Male		Female		Total^a^		% Female Pregnant
<1	290	(45.8%)	343	(54.2%)	633	(100.0%)	0.0%
	3.8%		3.6%		3.7%		
1-4	872	(51.6%)	819	(48.4%)	1691	(100.0%)	0.0%
	11.5%		8.6%		9.9%		
5-17	1242	(50.2%)	1232	(49.8%)	2474	(100.0%)	1.9%
	16.4%		12.9%		14.5%		
18-64	4694	(41.7%)	6571	(58.3%)	11265	(100.0%)	4.2%
	62.0%		69.0%		65.9%		
>65	472	(45.6%)	563	(54.4%)	1035	(100.0%)	0.0%
	6.2%		5.9%		6.1%		
Total	7570	(44.3%)	9528	(55.7%)	17098	(100.0%)	3.2%
	100.0%		100.0%		100.0%		

a 1,370 consultations out of 18,468 had missing gender information; thus, only data with gender information (17098) were analyzed.

Under the subsection entitled “Health Events and Procedure,” a total of 13,738 health events reported by EMTs were analyzed (Table 2). After excluding the high proportion of “other diseases, not specified” (56.5%), the majority of total health events were due to minor injuries (9.8%), acute watery diarrhea (9.4%), malaria (6.6%), ARIs (6.0%), and skin diseases (3.4%). Acute watery diarrhea (18.7%), ARIs (12.6%), and malaria (9.2%) were more common in children under-five, whereas minor injury (10.8%) was most common in patients aged five-years and older.

**Table 2.** Health Events and Procedures Done at EMTs during Cyclone Idai

		Age <5		Age ≥5		Total		P Value
		N	%	N	%	N	%	
Health Events								
Trauma	Major Head/Spine Injury	0	0.0%	12	0.1%	12	0.1%	
	Major Torso Injury	1	0.0%	6	0.1%	7	0.1%	
	Major Extremity Injury	4	0.2%	43	0.4%	47	0.3%	
	Moderate Injury	16	0.8%	181	1.6%	197	1.4%	.004
	Minor Injury	96	4.5%	1249	10.8%	1345	9.8%	.000
	Subtotal for Trauma	117	5.5%	1491	12.8%	1608	11.7%	
		[7.3%]		[92.7%]		[100.0%]		
Infectious Disease	Acute Respiratory Infection	268	12.6%	562	4.8%	830	6.0%	.000
	Acute Watery Diarrhea	398	18.7%	893	7.7%	1291	9.4%	.000
	Acute Bloody Diarrhea	39	1.8%	206	1.8%	245	1.8%	
	Acute Jaundice Syndrome	1	0.0%	4	0.0%	5	0.04%	
	Suspected Measles	0	0.0%	0	0.0%	0	0.00%	
	Suspected Meningitis	2	0.1%	4	0.0%	6	0.04%	
	Suspected Tetanus	0	0.0%	7	0.1%	7	0.1%	
	Acute Flaccid Paralysis	0	0.0%	1	0.0%	1	0.01%	
	Acute Hemorrhagic Fever	1	0.0%	2	0.0%	3	0.02%	
	Fever of Unknown Origin	52	2.4%	111	1.0%	163	1.2%	.000
	Subtotal for Infectious Disease	761	35.7%	1790	15.4%	2551	18.6%	
		[29.8%]		[70.2%]		[100.0%]		
Additional	Malaria	197	9.2%	708	6.1%	905	6.6%	.000
	Subtotal for Additional	197	9.2%	708	6.1%	905	6.6%	
		[21.8%]		[78.2%]		[100.0%]		
Emergency	Surgical Emergency (Non-Trauma)	7	0.3%	93	0.8%	100	0.7%	.018
	Medical Emergency (Non-Infectious)	33	1.5%	111	1.0%	144	1.0%	.000
	Subtotal for Emergency	40	1.9%	204	1.8%	244	1.8%	
		[16.4%]		[83.6%]		[100.0%]		
Other Key Diseases	Skin Diseases	109	5.1%	362	3.1%	471	3.4%	.000
	Acute Mental Health Problem	6	0.3%	88	0.8%	94	0.7%	.014
	Obstetric Complications	6	0.3%	54	0.5%	60	0.4%	
	Severe Acute Malnutrition	23	1.1%	15	0.1%	38	0.3%	.000
	Other Diagnosis, Not Specified Above	873	40.9%	6894	59.4%	7767	56.5%	.000
	Subtotal for Other Key Diseases	1017	47.7%	7413	63.9%	8430	61.4%	
		[12.1%]		[87.9%]		[100.0%]		
Total for Health Events		2132	100.0%	11606	100.0%	13738	100.0%	
		[15.5%]		[84.5%]		[100.0%]		
**Procedure**	Major Procedure	5	5.0%	59	4.0%	64	4.0%	
	Limb Amputation Excluding Digits	0	0.0%	9	0.6%	9	0.6%	
	Minor Surgical Procedure	96	95.0%	1187	79.6%	1283	80.6%	.000
	Normal Vaginal Delivery			95	6.4%	95	6.0%	
	Caesarean Section			34	2.3%	34	2.1%	
	Obstetrics Other			107	7.2%	107	6.7%	
Total for Procedure		101	100.0%	1491	100.0%	1592	100.0%	
		[6.3%]		[93.7%]		[100.0%]		

Note: P Value derived from Chi square test.

Abbreviation: EMT, Emergency Medical Team.

Moderate injury (P = .004), minor injury (P <.001), surgical emergency problem (P = .018), medical emergency problem (P <.001), and acute mental health problem (P = .014) were significantly higher in five-and-over patients compared to under-five patients, whereas acute watery diarrhea (P <.001), ARIs (P <.001), malaria (P <.001), and skin diseases (P <.001) were significantly higher in under-five patients.

Minor surgical procedures accounted for 80.6% of the 1,592 procedures performed.

**Figure 2.**
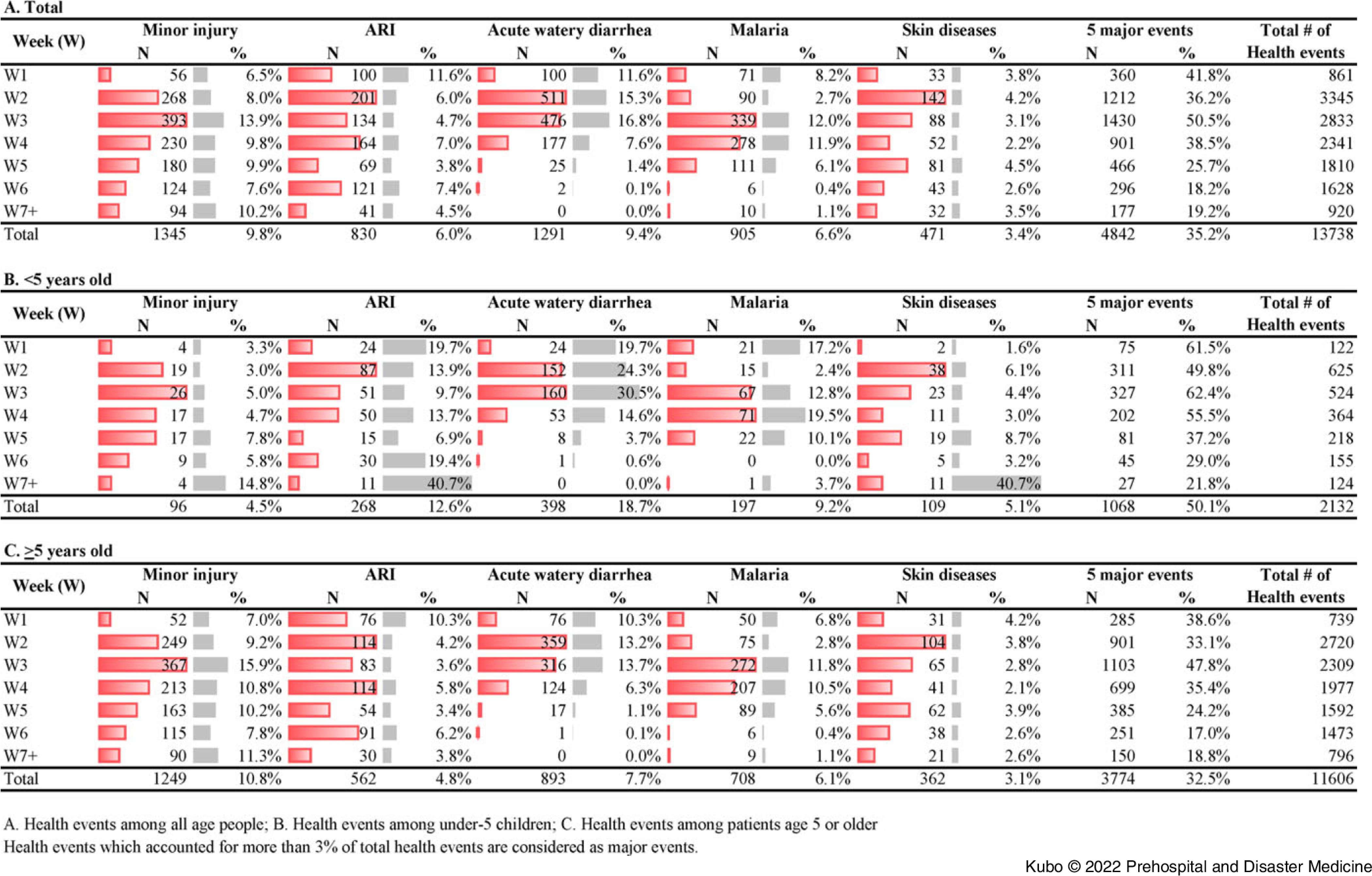
Distribution of Five Major Health Events by Period.

Figure 2 shows the weekly distribution of the top five events (minor injury, acute watery diarrhea, ARIs, malaria, and skin diseases). The number of these events peaked between Week 2 and Week 4 of the response. In terms of the proportion of health events to total consultations in the corresponding weeks, for under-five patients, minor injury (14.8%), ARI (40.7%), and skin diseases (40.7%) accounted for the highest proportions of total consultations in Weeks 8-16 of the response. This pattern was not observed for patients aged five-years and older.

Analysis of the “Outcome” and “Protection” subsections showed that more than one-half (58.7%) of consultations ended in discharge without follow up, while 16.1% required discharge with follow up. Twenty-five (0.04%) patients were reported to have died. Rehabilitation was required for 247 patients (1.3%). Additionally, EMTs reported 80 (1.7%) vulnerable children and 99 vulnerable adults (0.8%); Table 3.

**Table 3.** Outcome of consultation during Cyclone Idai

Outcome^a^	N	%
Discharge without Follow Up	10843	58.7%
Discharge with Follow Up	2979	16.1%
Discharge Against Medical Advice	48	0.3%
Referral	972	5.3%
Dead on Arrival	6	0.03%
Death within Facility	19	0.1%
Requiring Rehabilitation	247	1.3%
**Protection**		
Vulnerable Child^b^	80	1.7%
Vulnerable Adult^c^	99	0.8%

a % of among total number of consultations.

b Among 0–17-year-old children.

c Among over 18-year-old patients.

**Figure 3.**
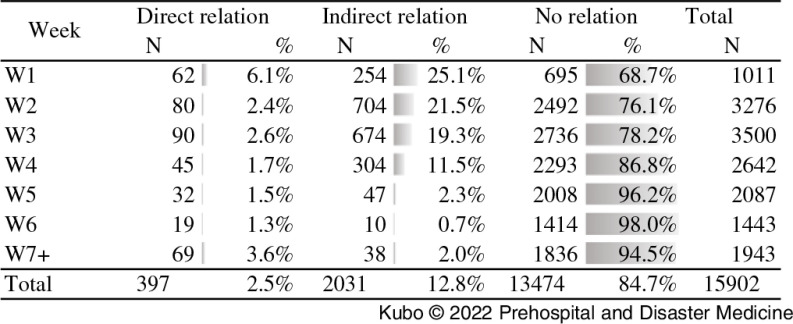
Patient Consultation in Terms of Relation to the Disaster.

Figure 3 shows the analysis of the “Relation” subsection. Out of 15,902 reported events with information available for the relationship to the disaster event, 2.5%, 12.8%, and 84.7% were directly related, indirectly related, and non-related, respectively, to the disaster event. During the response period, the proportion of non-disaster-related events increased from 68.7% to 94.5%.

## Discussion

In this section, the current study is discussed in accordance with four pillars including operational achievements of the EMT MDS, key finding, challenges and opportunities, and limitation of studies.

### Operational Achievements

First, the EMT MDS allowed health data to be collected from the onset of the response, with a total of 18,468 consultations recorded from all EMTs. It is very difficult to manage and report data during a disaster response, but this practice must be fostered. The ease of application of the EMT MDS and the robust logistical capacity of the EMTs proved to be key elements that allowed data to be collected even during the acute phase of the disaster.

Second, despite facing challenges that will be mentioned later during the response phase, the EMTCC was able to generate systematic reports on a daily basis. This was possible because the WHO has standardized the daily report form in both paper and csv data formats, facilitating the data analysis. Also, remote support from an off site data management support team played an important role, particularly for data input, since some EMTs submitted their daily reports in the paper form.

Third, the summary provided a better understanding of the evolving situation and enabled the EMTCC to make timely decisions. For example, it was previously reported that women experienced barriers limiting their access to medical care during emergencies due to religious or cultural issues.^9^ However, the EMT MDS showed that more than one-half of the consultations were for females (Table 1), indicating that female patients had good access to EMT services during this response. Evidence indicating fair access to EMT services is crucial during an operation. As another example of the collected data enabling timely decision making, consultations directly related and not related to the cyclone were found to show an inverse correlation over time, prompting the EMTCC to suggest that the international EMTs prepare their exit strategies as early as April 19 (Figure 3). Furthermore, greater access to data from EMTs and a proper level of analysis enabled and improved coordination with wider stakeholders and prioritization of needs.^10^ The willingness and leadership of the government of Mozambique also played an important role in the success of the disaster response and data management, including information sharing.^11^

### Key Findings

Key findings from the EMT MDS data collected during the response to Cyclone Idai are as follows.

Women were more likely than men to visit EMTs. This is consistent with other study results derived from analysis of the 2018 response following heavy rain in West Japan and the 2019 response to an earthquake in Kumamoto and Hokkaido, Japan.^12^ Outside of statistical gender considerations, this finding could reflect that the majority of men are either working to remediate the disaster or at work during the day whereas women are more likely to stay with family during a disaster and therefore more likely to have a chance to visit EMTs. This finding supports the importance of gender balance within the EMT personnel. This could be especially emphasized when providing care to Muslim countries, since a Muslim female health care provider would be the first choice for a Muslim female patient, followed by a non-Muslim female health care provider.^13^ The current analysis showed that normal vaginal delivery, cesarean section, and other obstetric procedures accounted for 15% of the total procedures performed at EMTs. Ninety-four live births were recorded, and 3.2% of the total consultations provided for women were pregnancy-related. This re-emphasizes the importance of ensuring access to obstetric and gynecologic care as part of the core services provided by EMTs, even during disaster events.^3^ Pregnant women gave birth at the EMT facilities during the first few weeks of the disaster response, as many health facilities were destroyed or non-functional,^14^ whereas no birth was reported at an EMT facility after Week 7 of the response. This shows the EMTs can play an important role as substitute health facilities but do not interfere with the existing referral pathways when the latter are re-established. This is consistent with previous study examining the utilization rate of health facilities versus EMTs by Japanese pregnant women.^15^ Minor injuries were more than twice as common in those over the age of five years compared to children under the age of five years. This is consistent with the findings obtained for the response to Typhoon Hayian, which struck the Philippines in 2013, and is thought to suggest that smaller children are more closely protected by their caregivers.^16^ Medical problems such as acute watery diarrhea and ARIs were the most common among children under five years. Acute watery diarrhea decreased steadily after Week 3 of the response, which could reflect the effect of the disease surveillance system established by the MOH of Mozambique to detect and respond to any outbreak of infectious disease, particularly cholera. Overall, it is important to note that the proportion of EMT consultations for non-disaster-related events steadily increased over time (Figure 3). Definitions of relation to disaster on the MDS, including (1) directly related to disaster (patient visit with injury or illness directly caused by a disaster; (2) indirectly related to disaster (patient visit with injury or illness caused or worsened by situational change after a disaster such as ARI, diarrhea, skin disease, injury by debris during clean-up); and (3) not related to disaster (patient visit with health problem not directly/indirectly related to the emergency event such as non-communicable diseases without acute exacerbation or essential medication loss, cancer, or appendicitis), are very practical but allow changes in situation or status to be detected in a timely manner and used to guide the demobilization timing of EMTs. Additionally, a high proportion of consultations were classified as having no relation to the disaster. This shows that during the acute phase of a response, EMTs substitute for regular health facilities experiencing disaster-limited functionality.

### Challenges and Opportunities

The challenges and technical gaps identified during the response are as follows.

First, there were some issues related to the completeness and accuracy of data. As this was the first time the EMT MDS was activated for a large international-scale response, the identified gaps might reflect a lack of pre-training. Indeed, only a very brief explanation and minimal guidance were provided to EMTs during a single EMTCC meeting. As an example of an issue with the completeness or accuracy of data, 94 live births were reported in the “Daily Summary” section (Figure 1) while 133 vaginal delivery and cesarean sections were reported in “Procedure” section (Table 2). It was impossible to determine whether the 35 discrepant cases were stillbirths or whether EMTs failed to report a birth from caesarean section as a live birth. This strongly suggests that pre- and real-time training for data collection is critical during a disaster response. The second identified challenge was the need for dedicated human resources with good knowledge of the EMT MDS system for joint management of EMT data collection and EMTCC data management. Third, due to lack of internet access, lack of experience in using the EMT MDS, and/or not perceiving the EMT MDS as a priority, some teams provided daily reports only occasionally. It proved challenging to use the EMT MDS in areas with poor internet connection, but the self-sufficiency of EMTs in their logistic capacity and offline data recording should allow for regular reporting to the coordination body. The time for daily submission was reported to be challenge, given the way each EMT set its daily operational cycle. As a result, during the response, the EMTCC suggested that each EMT should decide their own cut-off time for reporting, as long as the interval was 24 hours and two feedback summary reports were produced, with the second one including data on any delayed submission. Recognizing that the above setup is not ideal, it is proposed that timely data submission and compliance should be pursued through increased awareness of the EMT reporting system and dedicated training.

Points to be considered for further use of the MDS are as follows. First, more than one-half of consultations were reported as “other diseases, not specified” suggesting that some important health events may have not been captured or issues due to the definitions utilized and the way the data were reported. Therefore, regular analysis and discussions with the medical teams should be undertaken to determine additional health problems that should be included in the EMT MDS form as additional line items based on the local context. In the current case, only malaria was recorded as an “Additional” disease, as suggested by the EMTCC. Second, it is noted that many cholera and malaria cases were reported under the “Needs and Risks” section, which overlapped with items already included under the “Health Events and Procedure” section. Additionally, the information provided in the “Needs and Risks” section varied greatly. This suggests that more specific guidance should be provided about the importance and use of this part of the EMT MDS form. Third, given the above, it is believed that a survey should be undertaken among EMTs and other stakeholders who used the EMT MDS during the Cyclone Idai response, including questions about the usefulness and appropriateness of items and challenges encountered while using the EMT MDS form. This could help the current team further improve the MDS form and facilitate adoption during future health emergency responses.

The EMT MDS daily reporting system aims to improve the standardization of health data collection and analysis during emergency medical responses. Although the EMT MDS is currently well-known among classified EMTs, awareness should be increased among wider stakeholders and further training on the system should be undertaken. This will allow the MOH and its counterpart agencies to access the information they need to allocate their resources during the initial phase of an emergency, to ensure continuation of quality care to benefit the health of population affected by the disaster.

## Limitations

The current analysis has a few limitations that worth mentioning. First, due to the fact that it is the first activation of MDS in international-response scale, EMTs involved in this response lack adequate training of MDS use as aforementioned. Thus, it may have caused the under-reporting or misreporting of the health dataset. Second, some teams could only provide aggregated data but not individual-level data due to a lack of experience in handling the data. Therefore, only aggerated data were used in the analysis of the current study. Proper reporting will enable individual-level analysis, maximizing the potentials of data utilization in the future.

## Conclusion

The EMT MDS daily reporting system was activated for the first time on a large international-scale disaster in March 2019 during the response to Cyclone Idai in Mozambique, and a total of 18,468 consultations were provided by 13 EMTs from March 27 through July 12, 2019. Women accounted for a higher percentage of consultations than men. Minor injuries, acute watery diarrhea, malaria, ARIs, and skin diseases were most consulted. It was noticed that 85% of the consultations were non-disaster-related events. Despite numerous challenges, the EMT MDS reporting system was supportive for the response and coordination of EMTs. The contributions made by the MOH of Mozambique, the cooperation among different EMTs, and the inclusion of dedicated technical support allowed the EMT MDS to be successfully implemented during the Cyclone Idai response in March 2019.

## Supplementary material

For supplementary material accompanying this paper visit https://doi.org/10.1017/S1049023X22001406. click here to view supplementary material
