# Supplementary material for: First Activation of the WHO Emergency Medical Team Minimum Data Set in the 2019 Response to Tropical Cyclone Idai in Mozambique
Source: Prehosp Disaster Med. 2022 Nov 3;37(6):727–34. doi: 10.1017/S1049023X22001406 (PMC9726470; doi:10.1017/S1049023X22001406)
Supplement: Supplementary file 1 [file S1049023X22001406sup001.pdf]

EMT-MDS Daily Reporting Form(Ver1.0)

Team information

a	Organization name:				
b	Team name:				
c	Type 1 mobile	Type 1 fixed	Type 2	Type 3	Specialized cell
d	Contact Person(s) name(s):				
e	Phone No.:				
f	Email:				
g	Estimated date of departure (dd/mm/yyyy):				

h	Date of activity (dd/mm/yyyy):
i	Time of reporting (dd/mm/yyyy/hh:mm(24h)):
	**Location †**
j	Province (admin1)
k	District (admin2)
l	Village etc. (admin3)
m	Facility name:
n	Geo-tag ____________ (Lat) ____________ (Long)

† Submit one form per one activity day and location. For Admin 1 = e.g. State, Province, Governorate; Admin 2 = e.g. County, District, City, Municipality; Admin 3 = e.g. Sub-district, Village, Payam.

Daily Summary

Number of patient / Bed Count

o	Patients	Total Number of new consultation ‡	
p	Patients	New admission (=MDS40)	
q	Patients	Live Birth	
r	Bed	Total bed capacity	
s	Bed	Empty inpatient bed (Non-ICU)	
t	Bed	Empty Intensive Care Unit Bed (ICU)	
u			

MDS statistics ‡

No			
36	Outcome	Discharge without medical follow-up	
37	Outcome	Discharge with medical follow-up	
38	Outcome	Discharge against medical advice	
39	Outcome	Referral	
41	Outcome	Dead on arrival	
42	Outcome	Death within facility *	
43	Outcome	Requiring long term rehabilitation *	
44	Relation	Directly related to event	
45	Relation	Indirectly related to event	
46	Relation	Not related to event	
47	Protection	Vulnerable child *	
48	Protection	Vulnerable adult *	
49	Protection	Sexual Gender Based Violence (SGBV) *	
50	Protection	Violence (non-SGBV) *	

‡ Consider 24 hours period from midnight or other agreed cut off time for reporting. MDS statistics report outpatient consultations, inpatient admissions, as well as preformed procedures (MDS No.30-35), outcomes (MDS 36-43) and contextual issues (MDS No.44-50) newly counted during the reported period. MDS No.43 is a subset of MDS No.37-38. MDS No.47-50 are a subset of MDS No.53.

Demographic – MDS statistics

No		Age Categories	<1	1-4	5-17	18-64	65-	Total
1	Sex & Age	Male						
2	Sex & Age	Female non-preg.						
3	Sex & Age	Female pregnant						

Health Events and Procedure – MDS statistics

No		Health Events	<5	>=5	Total
4	Trauma	Major head / spine injury			
5	Trauma	Major torso injury			
6	Trauma	Major extremity injury			
7	Trauma	Moderate injury			
8	Trauma	Minor injury			
9	Infectious disease	Acute respiratory infection			
10	Infectious disease	Acute watery diarrhea			
11	Infectious disease	Acute bloody diarrhea			
12	Infectious disease	Acute jaundice syndrome			
13	Infectious disease	Suspected measles			
14	Infectious disease	Suspected meningitis			
15	Infectious disease	Suspected tetanus			
16	Infectious disease	Acute flaccid paralysis			
17	Infectious disease	Acute haemorrhagic fever			
18	Infectious disease	Fever of unknown origin			
19	Additional §	Malaria			
20	Additional §				
21	Additional §				
22	Additional §				
23	Emrg.	Surgical emergency (Non-trauma)			
24	Emrg.	Medical emergency (Non-infectious)			
25	Other key diseases	Skin disease			
26	Other key diseases	Acute mental health problem			
27	Other key diseases	Obstetric complications			
28	Other key diseases	Severe Acute Malnutrition (SAM) *			
29	Other key diseases	Other diagnosis, not specified above			
		**Procedure**	**<5**	**>=5**	**Total**
30	Procedure	Major procedure (excluding MDS31)			
31	Procedure	Limb amputation excluding digits *			
32	Procedure	Minor surgical procedure			
33	Procedure	Normal Vaginal Delivery (NVD)			
34	Procedure	Caesarean section			
35	Procedure	Obstetrics others			

Needs and Risks

Free text reporting to EMTCC / MOH on the following issues.

No			
51	Immediate report	Unexpected death *	☐
52	Immediate report	Notifiable disease *	☐
53	Immediate report	Protection issues #	☐
54	Immediate report	Critical incident to EMT and/or community	☐
55	Immediate report	Any other issue requiring immediate reporting	☐
56	Community Risks	WASH	☐
57	Community Risks	Community / suspected over infectious disease	☐
58	Community Risks	Environmental risk / exposure	☐
59	Community Risks	Shelter / Non food items	☐
60	Community Risks	Food insecurity	☐
61	Operational constraints	Logistics / operational support	☐
62	Operational constraints	Supply	☐
63	Operational constraints	Human resources	☐
64	Operational constraints	Finance	☐
65	Operational constraints	Others	☐

Detailed comment for (No.)

Detailed comment for (No.)

Detailed comment for (No.)

Detailed comment for (No.)

* Line list (including detailed information) should be submitted with this MDS form to relevant authorities. § Additionals are used for context specific reporting items indicated by the relevant authorities e.g. Malaria / Dengue / TB / Leptospirosis / Rabies / Hazmat etc. # Protection issues to be reported confidentially to appropriate authority or protection cluster in locally agreed manner.
